# Dysregulated circRNA expression profile and associated miRNA sponging in abnormal lung development in congenital diaphragmatic hernia

**DOI:** 10.1002/ctm2.70752

**Published:** 2026-09-11

**Authors:** Marietta Jank, Arzu Ozturk Aptekmann, Rina Tanaka, Muntahi Mourin, Nolan De Leon, Matthew Kraljevic, Daywin Patel, Wai Hei Tse, Claire McCallum, Richard Wagner, Shana Kahnamoui, Yuichiro Miyake, Athanasios Zovoilis, Takashi Doi, Michael Boettcher, Richard LeDuc, Richard Keijzer

**Affiliations:** ^1^ Section of Pediatric Surgery Department of Surgery Max Rady College of Medicine Rady Faculty of Health Sciences University of Manitoba Children's Hospital Research Institute of Manitoba Winnipeg Manitoba Canada; ^2^ Department of Pediatric Surgery University Medical Center Mannheim Heidelberg University Mannheim Germany; ^3^ Department of Pediatric Surgery Kansai Medical University Osaka Japan; ^4^ Department of Biochemistry and Medical Genetics University of Manitoba and Children's Hospital Research Institute of Manitoba Winnipeg Manitoba Canada; ^5^ Department of Pediatric Surgery University of Leipzig Leipzig Germany; ^6^ Department of Pediatric General and Urogenital Surgery Juntendo University School of Medicine Tokyo Japan

**Keywords:** biomarkers, circular RNAs, diaphragmatic, hernia, prenatal care, pulmonary hypoplasia, RNA

## Abstract

**Rationale:**

Circular RNAs (circRNAs) are stable, tissue‐ and developmental‐stage‐specific regulators of gene expression and candidate disease biomarkers. Their expression profile in abnormal lung development in congenital diaphragmatic hernia (CDH) is unknown.

**Objective:**

To evaluate circRNA expression profile in CDH‐associated abnormal lung development.

**Methods:**

We profiled circRNAs in rat CDH and control lungs at embryonic day (E)15 and E21 by microarray. We validated identified circRNAs using back‒splice junction amplicon sequencing, RT‐qPCR and in situ hybridisation. We modified a circRNA function prediction tool to predict circRNA::micro(mi)RNA::messenger(m)RNA interactions and compared these with Oxford Nanopore RNA sequencing and existing human CDH datasets.

**Measurements and main results:**

Microarrays revealed a unique circRNA biosignature during CDH lung development. CircAnp32e was expressed in a sex‐specific and spatiotemporal expression pattern in the epithelium at E15. The predicted mature sequence of circAnp32e overlapped 90% with its human orthologue. circRNA::miRNA::mRNA interaction networks at E15 and E21 revealed enrichment in inflammation/infection, smooth muscle cell function, cell proliferation/cell cycle regulation and response to hypoxia pathways. Parental genes of differentially expressed circRNAs at E15 enriched pathways linked to cell proliferation/cell cycle/cancer, while at end‐gestation, inflammation and cardiovascular processes were also overrepresented. Rat and human CDH lungs showed overlapping pathways with additional enrichment for RNA processing and protein binding/modification in humans. In a human bronchial epithelial (BEAS‐2B) nitrofen‐injury model, *ANP32E* and circANP32E were downregulated, and the predicted let‐7 target was significantly dysregulated.

**Conclusion:**

A unique circRNA signature during abnormal lung development in CDH may mediate inflammatory responses, smooth muscle cell function and cell proliferation regulation via miRNA sponging. Overlap of downstream pathways in rat and human CDH suggests conserved functions across species. This circRNA biosignature defines strong candidate biomarkers for CDH and a basis for future prospective prenatal investigation.

## Graphical abstract

Circular RNAs (circRNAs) are stable, stage‐ and tissue‐specific regulators of gene expression warranting their study in abnormal fetal lung development in congenital diaphragmatic hernia (CDH). We present a CDH‐specific circRNA biosignature in the nitrofen rat model (E15/E21) using orthogonal validation with human comparison and discovered conserved circRNA::miRNA::mRNA networks linked to hypoxia/inflammation, epithelial–mesenchymal transition/endothelial‒mesenchymal transition (EMT/EndoMT) and smooth‐muscle pathways, including sex‐specific expression and epithelial localisation of circAnp32e.

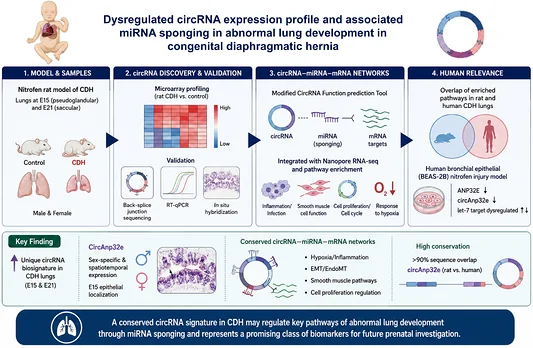

## 1 INTRODUCTION

Lung development requires a delicate balance of cell proliferation, migration and differentiation.^1^ This balance is disturbed in congenital diaphragmatic hernia (CDH) causing cardiopulmonary hypoplasia, pulmonary hypertension and a diaphragmatic defect.^2^

Circular RNAs (circRNAs) regulate gene expression and cellular processes in development and disease.^3^, ^4^ circRNAs have a covalently closed‐loop configuration generated by backsplicing, which provides them with remarkable stability.^3^ circRNAs can serve as micro(mi)RNA sponges, protein scaffolds and peptide templates via non‐canonical translation, while modulating transcription, alternative splicing and mRNA translation to regulate cell proliferation, apoptosis and stress responses.^3^

circRNAs show a tissue‐ and developmental stage‐specific expression making them attractive biomarkers for congenital anomalies. We previously published a unique circRNA biosignature in the lungs of deceased CDH cases at mid‐ and end‐gestation.^5^ We have published dysregulation of a circRNA derived from the parental gene *Tial1* in the nitrofen rat model for CDH at late gestation and established a modified bioinformatics pipeline to predict mature circRNA sequences and biological functions through miRNA sponging.^6^ Here, we hypothesised that circRNAs are dysregulated during abnormal lung development in CDH and act as miRNA sponges within conserved regulatory networks that may link environmental exposure to the inflammatory and epithelial–mesenchymal transition (EMT)‐related pathways during abnormal lung development.

## 2 METHODS

### 2.1 Samples

Procedures were approved by the Animal Research Review Committee (23‐026 [AC11831]) and human Research Ethics Board (HS15293 [H2012:134]) at the University of Manitoba. The nitrofen model was employed as previously described.^6^ Lungs were harvested at E15 and at E21 only from foetuses with confirmed diaphragmatic defects. Sex was determined using X‐ and Y‐specific primers (Table S1).

### 2.2 RNA isolation, RNase R treatment and DNA isolation

Total RNA was extracted from foetal lungs at E15 and E21, quantified and enriched for circRNAs by RNase R digestion. Genomic DNA isolation was performed with in‐column RNase A digestion. See Supporting Information for details.

### 2.3 Microarray expression of circRNAs

circRNAs were labelled using random primers and hybridised to Arraystar Rat Circular RNA Microarrays (Ref. AS‐S‐CR‐R‐V2.0, Arraystar Inc.). Microarray was performed on three control and three nitrofen‐induced biological replicates at E15 and E21 and each biological replicate was a pool of four to five foetal lungs from a single dam. See Supporting Information for details.

### 2.4 cDNA library generation, circRNA back‒splice junction detection, subcloning and Sanger sequencing

Primers were designed in‐house, and PCR was performed on diluted RNase R‐treated cDNA after cDNA synthesis. Plasmids from positive colonies were sequenced and analysed once ligation products were transformed and selected. See Supporting Information for details.

### 2.5 RT‐qPCR

Following primer optimisation and sequence verification, RT‐qPCR was performed on diluted cDNA (1:100 for parental genes and 1:10–1:20 for circRNAs). See Supporting Information for details (Table S2).

### 2.6 BaseScope in situ hybridisation

Sections were hybridised with BaseScope probes (Table S3) and amplified following a modified manufacturer's protocol (Table S4).

### 2.7 Oxford nanopore sequencing

RNA was extracted from three nitrofen induced and three control lungs at E15 and E21 as described above and sequenced. For a detailed protocol of the Oxford Nanopore sequencing experiment, see Table S5, S9 and Supporting Information.

### 2.8 Human bronchial epithelial cell (BEAS‐2B) nitrofen‐injury model and downstream target validation

To functionally interrogate the predicted circANP32E::let‐7::mRNA target axis in a human epithelial context, human bronchial epithelial cells (BEAS‐2B) were exposed to nitrofen or vehicle (dimethyl sulfoxide, DMSO) and harvested at 24  h. Cells were cultured according to manufacturer's recommendations and 1.2 × 10^5^ cells were cultured overnight and treated with 50 µM nitrofen for 24 h. Next, BEAS‐2B cells were transfected with a hsa‐let‐7b‐5p mimic, inhibitor (each at 25 µM) or a non‐targeting negative control. Total RNA was isolated using Direct‐zol RNA miniprep (Cat# R2071 Zymo Research) and expression levels of *ANP32E* parental and circANP32E and downstream target validations were determined (Supporting Table S2).

### 2.9 Bioinformatics analysis and modified CRAFT

Downstream targets as predicted by our modified CircRNA Function prediction Tool (CRAFT) pipeline were enriched for Gene Ontology (GO) and Kyoto Encyclopedia of Genes and Genomes (KEGG) pathways.^7^, ^8^ Pathways were compared with direct RNA sequencing and human CDH datasets.^5^ See Supporting Information for details.

### 2.10 Statistical analysis

We quantified in situ hybridisation signal per cell in four random areas with HALO (Indica Labs). Sample sizes are reported in each figure legend. RT‐qPCR was performed on individual foetal lungs from different dams to allow sex‐disaggregated analyses. Human bronchial epithelial (BEAS‐2B) experiments used independent biological replicates. Statistical analyses and data visualisation were performed using GraphPad Prism version 9.0.2 (GraphPad Software) and R version 4.3.2 (R Core Team, 2023).

## 3 RESULTS

### 3.1 circRNA expression profile at E15 and E21

**FIGURE 1:**
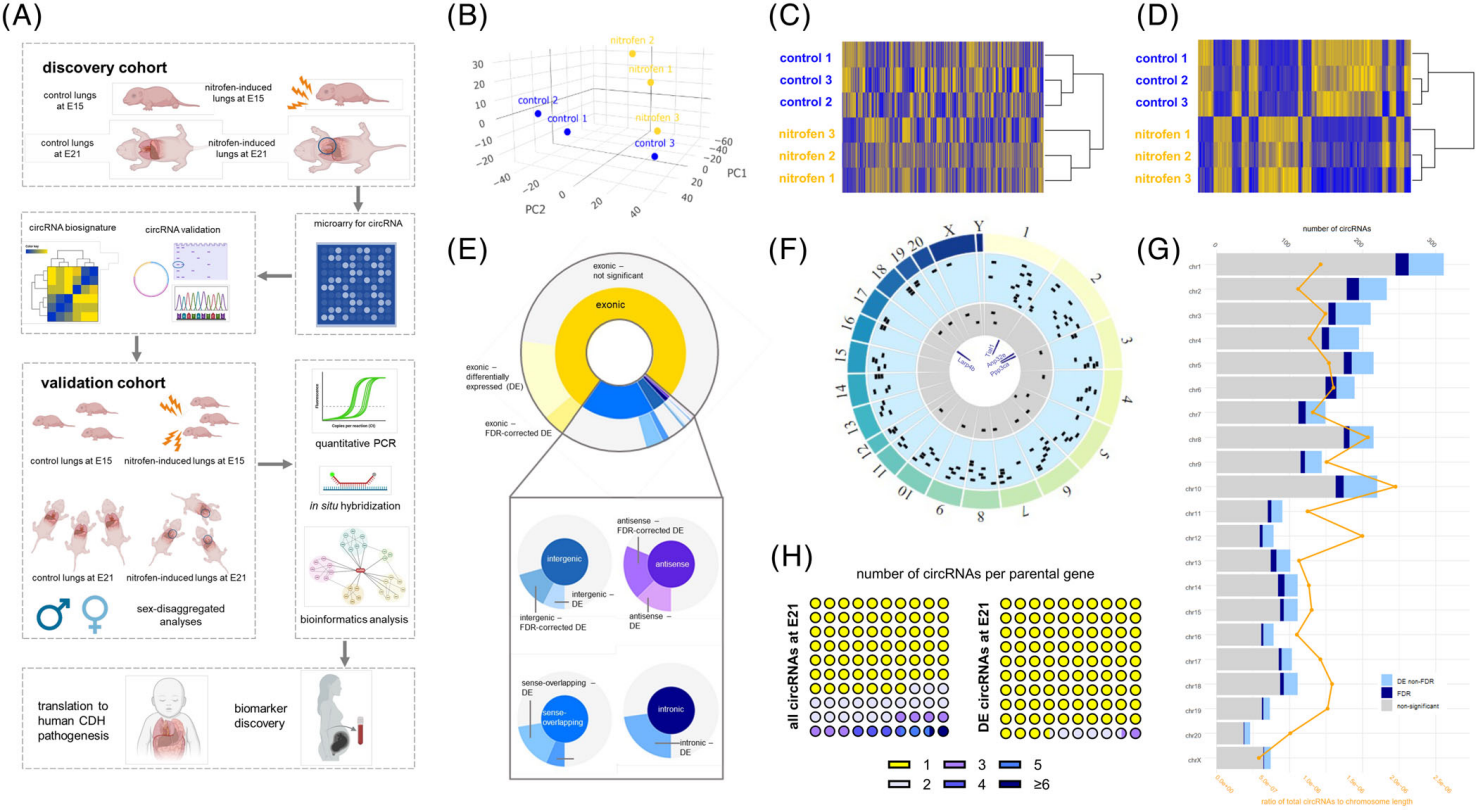
Unique biosignature for lung hypoplasia in congenital diaphragmatic hernia (CDH). Graphical abstract depicting the evaluation of the circular RNA (circRNA) profile in early and late stages of abnormal lung development in the nitrofen rat model for CDH using a microarray approach (A). Microarray was performed on three control and three nitrofen‐induced biological replicates (each consisting of four to five foetal lungs) per time point. Grouping of control and nitrofen‐induced lung samples at embryonic day (E)21 based on overall circRNA profile plotted on a principal component analysis (B) and heatmap of all detected circRNAs (C). A unique circRNA biosignature of differentially expressed (DE) circRNAs distinguishes control and CDH lungs at late gestation (D). Distribution of the circRNA type at E21 where most of the DE circRNAs (71%) are exonic or sense‐overlapping (22%) (E). Chromosomal location of the back‒splice junctions at E15 (inner circle) and E21 (outer circle) showing labelling of the circRNA candidates chosen for validation (F). The number of circRNAs across all chromosomes and the ratio of circRNAs relative to the chromosome length, indicating highest enrichment of chromosomes 10, 8 and 12 at E21 (G). Number of circRNAs per parental gene for all circRNAs and DE circRNAs at late gestation (H).

Microarray analysis of rat foetal left lungs detected 13 337 and 13 770 circRNAs at E15 and E21, respectively. Principal component analysis revealed clustering of nitrofen‐induced and control lung datasets at both stages (E15: Figure S1A and E21: Figure S1B). At E15, differentiation was visible between CDH and control lungs within differentially expressed (DE) circRNAs (Figure S1B,C). At E21, this distinction extended to all circRNAs, regardless of significance (Figure 1C,D). At E15, 119 circRNAs were significantly DE, with 22 passing Benjamini‒Hochberg adjustment. At E21, 681 were DE, and 163 remained significant after Benjamini‒Hochberg adjustment. Full microarray results for both embryonic stages are provided in Tables S6 and S7.

Exonic circRNAs accounted for 77% and 71% of DE circRNAs at E15 and E21, respectively. Sense‐overlapping circRNAs comprised 23% (E15) and 22% (E21), followed at E21 by intergenic (5%) and antisense (2%) circRNAs (Figures S1D,E). To identify chromosomal ‘hotspots’, we mapped circRNA coordinates and normalised by chromosome length. Similar patterns were evident at E15 (Figure S1E) and E21 (Figure 1G). Chromosome 1 showed the highest circRNA count at E21, while chromosomes 10, 8 and 12 had higher relative densities. Chromosome X had the lowest ratio (ratio = 4.85e^−7^). Multiple circRNAs originated from single parental genes (Figures 1H and S1F). At E21, *D* *cc* produced 12 isoforms. Among the DE circRNAs, nine had two isoforms from the same parental gene (*Dcc*, *Ddhd2*, *Ezh2*, *Nckap5*, *Ptk2*, *Rn18s*, *Stk39*, *Trps1* and *Wdr7*).

### 3.2 Validation of candidate circRNAs from parental genes Anp32e, Larp4b and Ppp3ca using subcloning and directional PCR

Based on microarray findings and relevant literature, we validated circRNAs derived from the parental genes *Anp32e*, *Ppp3ca*, *Larp4b* and *Tial1* ^6^ (Table 1).

**TABLE 1:** Selected circular RNA (circRNA) candidates.

circRNA	Chromosomal location	logFC at E15	*p*‐alue adjusted; *p*‐value	logFC at E21	*p*‐value adjusted *p*‐value	Parental gene	Gene function	Human orthologue conservation (%)
rno_circRNA_008695	2:(183476783‒183479457)	2.19	.068; not passed	‒3.18	2.09 e‐06; 8.68 e‐05	*anp32e*	Nuclear phosphoprotein and histone chaperone	90%
rno_circRNA_008976	1:(183012386‒183024629)	.88	.022; not passed	‒1.52	1.14 e‐08; 1.40 e‐06	*ppp3ca*	Serine/threonine protein phosphatase	78%
mmu_circRNA_42344	2:(225387868‒225405161)	‒.33	.502; not passed	1.97	2.30 e‐06; 9.42 e‐05	*tial1*	RNA‐binding protein	85%^6^
rno_circRNA_006702	17:(65036456‒68050063)	‒.22	.784; not passed	.81	.309; .510	*larp4b*	RNA‐binding protein	83%

**FIGURE 2:**
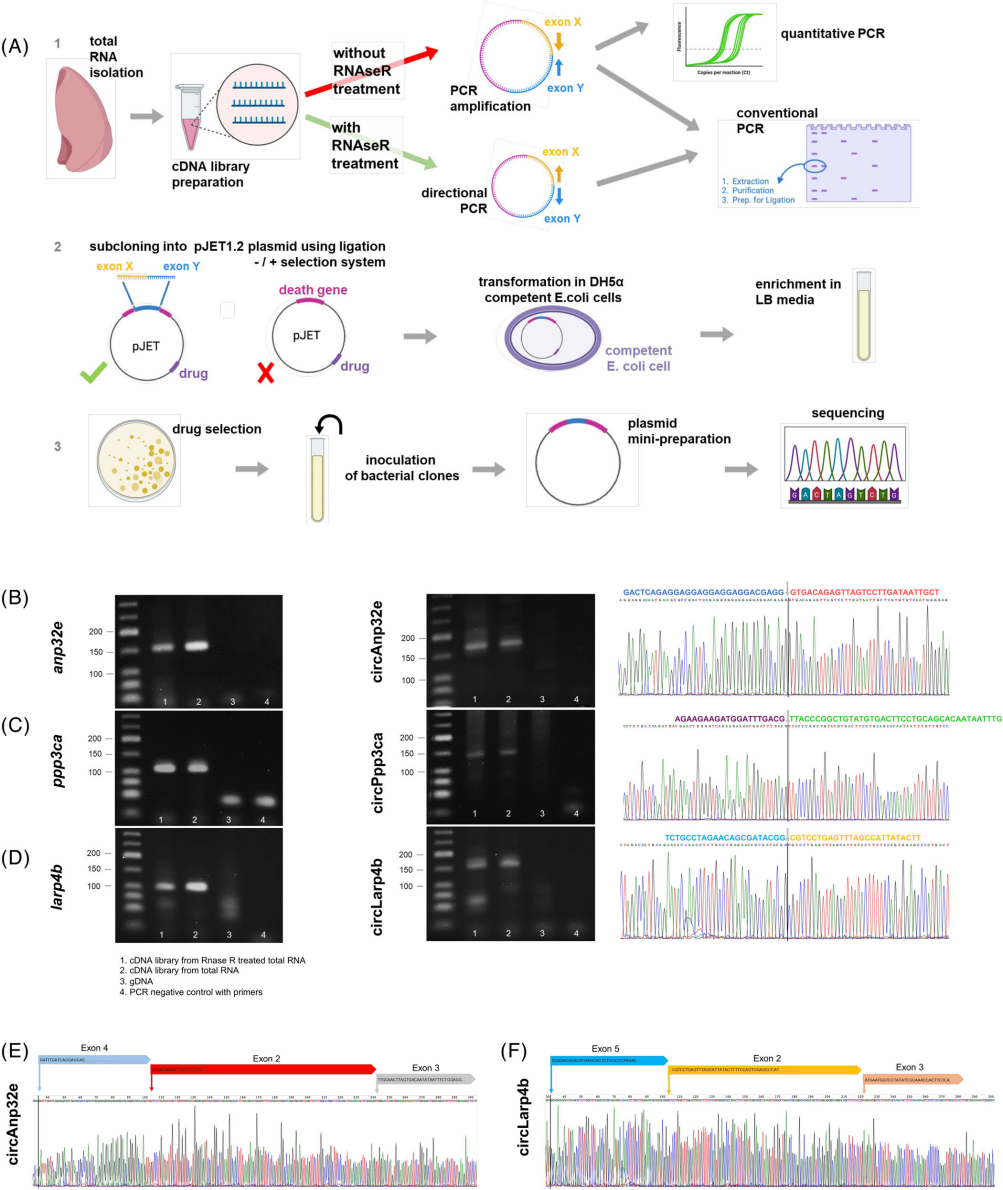
Validation of circAnp32e, circPpp3ca and circLarp4b by conventional PCR with convergent and divergent primers using pJET subcloning and amplicon sequencing. Total RNA was isolated and reverse transcribed; cDNA was treated with or without RNase R. Untreated samples enabled quantification by qPCR, while RNase R treatment depleted linear RNAs facilitating junction‐specific PCR and gel electrophoresis (A‐1). PCR products were subcloned into the pJET1.2 vector, transformed into competent cells and enriched in selective media (A‐2). Clones with inserts were selected via antibiotic resistance, followed by plasmid mini‐preparation and Sanger sequencing for validation (A‐3). This workflow was applied to candidate circRNAs and their parental genes using different experimental conditions. Back‒splice junctions were confirmed by Sanger sequencing of pJET1.2 subclones (*n* = 20 independent clones per circRNA). Expected amplicon sizes are annotated on each gel panel: circAnp32e product = 156 bp; *Anp32e* = 158 bp; circPpp3ca product = 131 bp; *Ppp3ca* product = 112 bp; circLarp4b product = 154 bp; *Larp4b* product = 104 bp. Representative gels are shown with the lanes: (1) cDNA synthesised from RNase R‐treated total RNA to selectively enrich for circRNAs, (2) cDNA synthesised from total RNA to account for linear RNA amplification and normalisation to a housekeeping gene for quantification by RT‐qPCR, (3) genomic DNA (gDNA) as a control for potential DNA contamination, and (4) PCR negative control without a template to assess non‐specific amplification. Divergent primers successfully amplified the circRNA derived from the parental genes *Anp32e*, *Ppp3ca* and *Larp4b* in RNase R‐treated cDNA but not in genomic DNA (B‒D, respectively). Directional PCR amplification detected the additional circRNA exonic junctions for circAnp32e and circLarp4b to prove the circular nature of the RNA molecule in rat lung tissues (E and F, respectively).

circAnp32e had the lowest logFC at E21 (‒3.18) and is linked to fibrosis, EMT and viral life cycles.^9^, ^10^ circAnp32e spans exons 4–2 on rat chromosome 2 and its back‒splice junction was confirmed using in‐house designed divergent primers on RNase R‐treated cDNA, followed by subcloning and Sanger sequencing (Figure 2B). Considering circRNAs are generated through post‐transcriptional backsplicing events and therefore absent in linear genomic DNA, back‒splice junction was only amplified in the cDNA pool with and without RNase R treatment (Figure 2B). We established directional PCR amplification using divergent primers validating alignment of exon 4–3–2 with head‐to‐tail splicing sites by Sanger sequencing (Figure 2E). This matches the 442‐nucleotide mature sequence of circAnp32e(2,3,4).1 annotated in the circAtlas database (rno‐Anp32e_0001; Figure S2A; accessed 28.7.2024).^11^

circPpp3ca was DE at E21, and aberrations in its parental gene have been linked to multiple congenital abnormalities.^12^ Its back‒splice junction composed of exons 10–7 on rat chromosome 2 was confirmed (Figure 2C; the form is also annotated in circAtlas; Figure S2B).^13^ Similarly, we validated the circRNA derived from *Larp4b*, an alternative splicing‐related RNA‐binding protein (RBP). circLarp4 expression correlates with non‐small cell lung cancer prognosis and suppresses cancer cell proliferation.^14^, ^15^ Sanger sequencing confirmed the back‒splice junction between exons 5 and 2 (Figure 2D) and exon alignment 5–2–3 (Figure 1F).

### 3.3 circRNA and parental gene expression profiles and spatiotemporal distribution of candidate circRNAs: circAnp32e, circLarp4b and circPpp3ca

**FIGURE 3:**
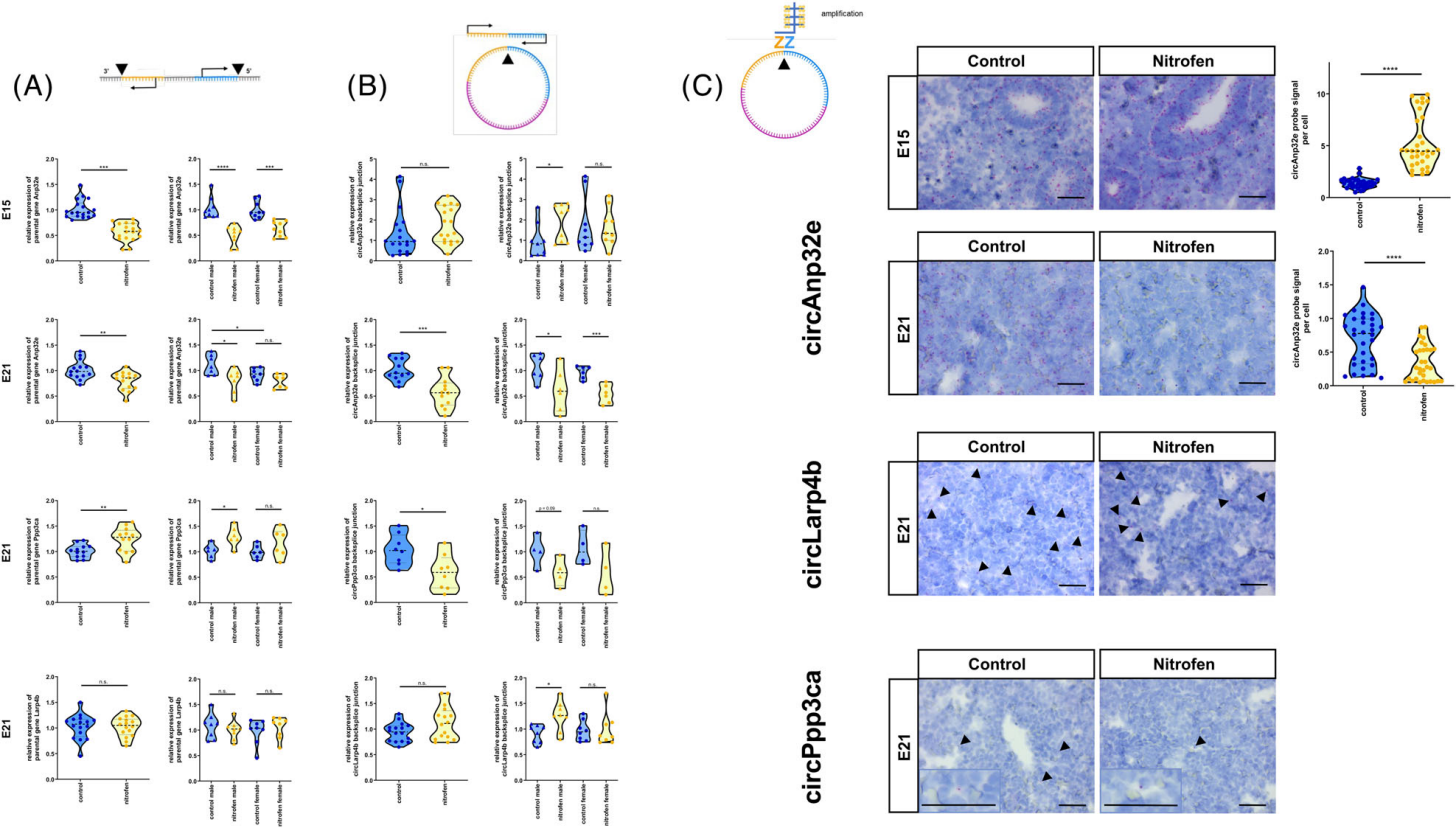
Sex‐ and developmental stage‐specific expression of selected circular RNA (circRNA) candidates. Linear mRNA transcripts are often co‐expressed with corresponding circRNAs, which in turn can regulate the expression of parental genes. Therefore, we performed quantitative PCR of both the parental gene (A) and circRNA (B). Each symbol represents one foetus. Sample sizes per nitrofen and control group were: (circ)Anp32e *n* = 18 at E15 and *n* = 12‒14 at E21; (circ)Ppp3ca *n* = 8‒12 and (circ)Larp4b *n* = 16 at E21; split approximately evenly between males and females. To refute a potential overestimation or false‐positive detection resulting from PCR‐based methods, we further confirmed the microarray results using in situ hybridisation (C). ^*^p < .05, ^**^p < .01, ^***^p < .001.

As circRNAs may co‐regulate their linear host transcripts,^3^ we quantified both parental genes and circRNAs from the same cDNA pool via RT‐qPCR (Figure 3A,B). *Anp32e* was downregulated in nitrofen lungs at E15 and E21; the interaction factor for sex in two‐way analysis of variance (ANOVA) was not significant (*p* = .75). RNA sequencing suggested *Anp32e* downregulation without reaching statistical significance (logFC = ‒3.5, *p* = .15; Figure S3B). At E15, overall circAnp32e expression was similar between controls and CDH samples (*p* = .22); however, expression was upregulated in male lungs (*p* = .034; Figure 3B). Consistent with our microarray studies, RT‐qPCR revealed a decreased circAnp32e expression in nitrofen‐induced lungs at E21 (*p* = .004). *Ppp3ca* was upregulated at E21, an effect driven by expression in male lungs (*p* = .006; Figure 3A). Consistent with microarray results, nitrofen lungs had decreased circPpp3ca expression at E21 (*p* = .016). Overall *Larp4b* and circLarp4b expression did not differ between control and nitrofen‐induced lungs, except for significantly elevated levels in male CDH lungs based on a sex‐disaggregated analysis (in line with the microarray data).

In situ hybridisation confirmed circAnp32e and circLarp4b expression (Figure 3C). The circAnp32e signal peaked in the epithelium of nitrofen lungs at E15. CircPpp3ca showed a specific but weak probe signal in both groups corroborating the high Cq values in RT‐qPCR; quantifying specific spatiotemporal patterns was not feasible (Figure 3C).

### 3.4 Bioinformatics analyses of predicted circRNA function through miRNA sponging in abnormal lung development

To explore circRNA function in abnormal lung development, we performed GO and KEGG pathway analyses of parental genes and circRNA::miRNA::mRNA targets at E15 and E21.

**FIGURE 4:**
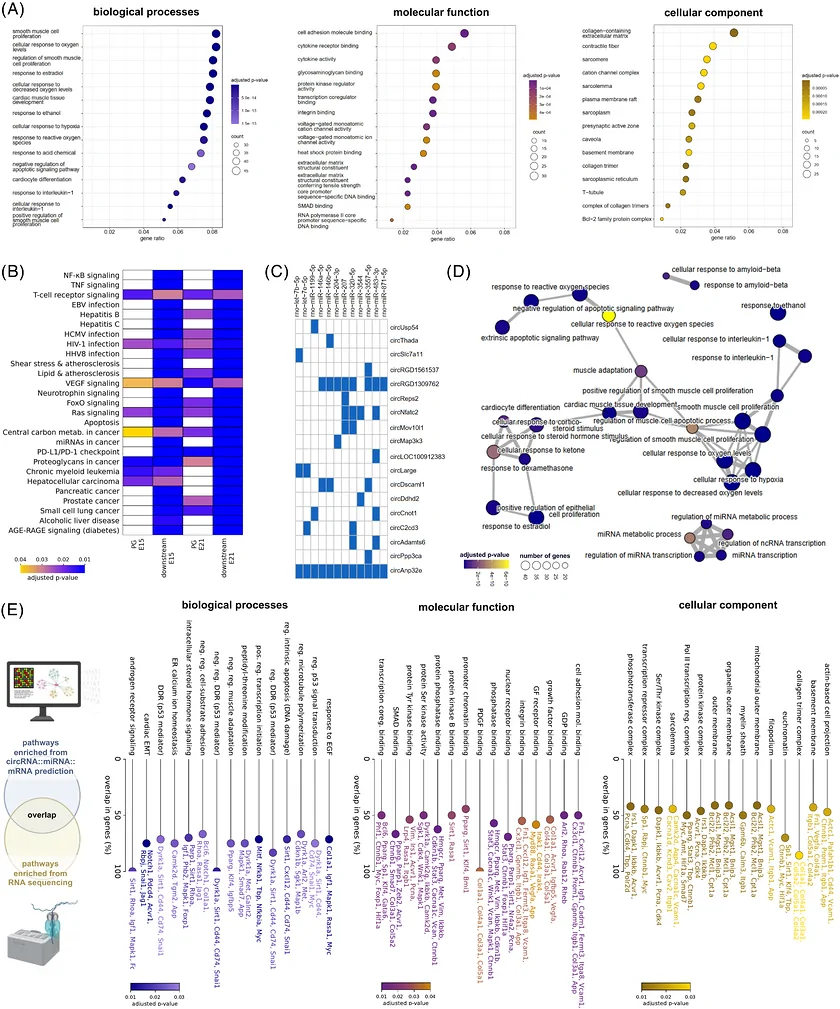
Enrichment for response to hypoxia, smooth muscle cell proliferation and inflammation. Enrichment of top 15 Gene Ontology (GO) terms from CRAFT‐predicted circRNA::miRNA::mRNA downstream targets at E21 focuses on muscle cells, extracellular matrix, response to hypoxia and binding (A). The heatmap compares the top 15 KEGG pathways enriched by parental genes and circRNA::miRNA::mRNA targets at E15 and E21, suggesting a substantial overlap in predicted biological functions by miRNA sponging at early and late gestation (B). Heatmap summarising circRNAs predicted to interact with the same miRNAs as circAnp32e (C). Pathway analyses of mRNA targeted by this circRNA network (D). Oxford Nanopore direct RNA sequencing was performed on *n* = 3 control and *n* = 3 nitrofen‐induced lungs at E21. The results were utilised to confirm predicted pathways and identify promising downstream targets. The plots show the overlap in significantly enriched GO terms, the proportion of differentially expressed (DE) genes identified through RNA sequencing and their respective names (E).

730 circRNA::miRNA::mRNA target genes at E15 and 583 at E21 enriched for various pathways, with complete overlap among the top 15 KEGG terms (Figure 4B). Additional terms related to vascular development and endothelial migration (biological processes), nuclear factor‐kappa B (NF‐κB) signalling (molecular function) and membrane and focal adhesion structures (cellular components) enriched at E15 (Figure S4B). At late gestation, 583 predicted downstream mRNA targets augmented biological processes linked to cellular response to hypoxia, regulatory networks in smooth muscle cell proliferation and cardiac muscle development, as well as apoptosis signaling and interleukin‐1 response. Enriched molecular functions included cytokine receptor binding and activity, cellular adhesion, and extracellular matrix function, while enriched cellular components comprised collagen matrix, muscle fibre components and membrane structure (Figure 4A).

The parental genes of DE circRNAs at E15 enriched for splicing/mRNA processing, and (mesenchymal/stem/neural crest) cell development as well as migration (Figure S4A). Enriched GO terms for molecular functions were predominantly binding related, while cellular components included RNA‐regulatory complexes (Figure S4A). While at E21, we found enriched GO terms related to muscle hypertrophy and a unique cluster around neuron development (Figure S4C). Both embryonic stages were associated with KEGG pathways related to inflammation and infection, as well as cancer, cell cycle regulation and proliferation (Figure 4B).

Next, we analysed miRNA sponging by circAnp32e (Figure S6D) and DE circRNAs with shared miRNAs (Figure 4C). CircAnp32e was predicted to interact with miRNAs known to influence lung development in CDH, including miRNA‐let‐7b‐5p and miRNA‐let‐7e‐5p (Figure S6D).^16^ The heatmap summarises circRNAs that were predicted to interact with the same miRNAs as circAnp32e; one of which is circPpp3ca (Figure 4C). Pathway analyses of mRNA targeted by this circRNA network revealed GO terms associated with smooth muscle and epithelial cell proliferation, apoptosis and miRNA transcription (Figure 4D).

Finally, validation using Oxford Nanopore RNA sequencing showed consistent overlap in enriched GO terms between the predicted circRNA::miRNA::mRNA targets and observed expression changes (Figures 4E and S7A). Not only did the enriched biological processes linked to apoptosis, EMT and muscle adaptation overlap, genes contributing to these pathways showed >60% similarity (Figure 4E). All enriched KEGG terms from the CRAFT‐based prediction and RNA sequencing presented a moderate positive correlation (Figure S7B).

### 3.5 Indications of overlap in circRNA profile and function between human and rat CDH at later stages of abnormal lung development

Given the evolutionary conservation of circRNAs, we compared expression profiles between rat CDH lungs at E21 and our previously published human CDH lung profiles at mid‐ and end‐gestation.^5^ Human CDH showed higher total circRNA enrichment for chromosomes 17 and 19 (Figure S5A,B) and DE circRNAs clustered on chromosome 1 (Figure S5C). Shared parental genes of DE circRNAs in rat and human CDH were associated with lung disease, embryonic development, fibrosis, proliferation and RNA/DNA regulation (Table S8).

Predicted circRNA::miRNA::mRNA targets demonstrated conserved enrichment in GO terms between the two species, including (1) cellular response to hypoxia and regulation of miRNA transcription (biological processes), (2) transcription factor binding and regulation of translation activity (molecular functions), and (3) chromosome‐associated components (cellular components). Shared KEGG terms enriched for inflammation and infection (e.g., NF‐κB and tumour necrosis factor [TNF] signalling, viral infections), regulation of cell cycle and proliferation, as well as cardiovascular pathways (Figure S6C). Interestingly, only human CDH showed enrichment for epigenetic pathways (Figure S6C).

**FIGURE 5:**
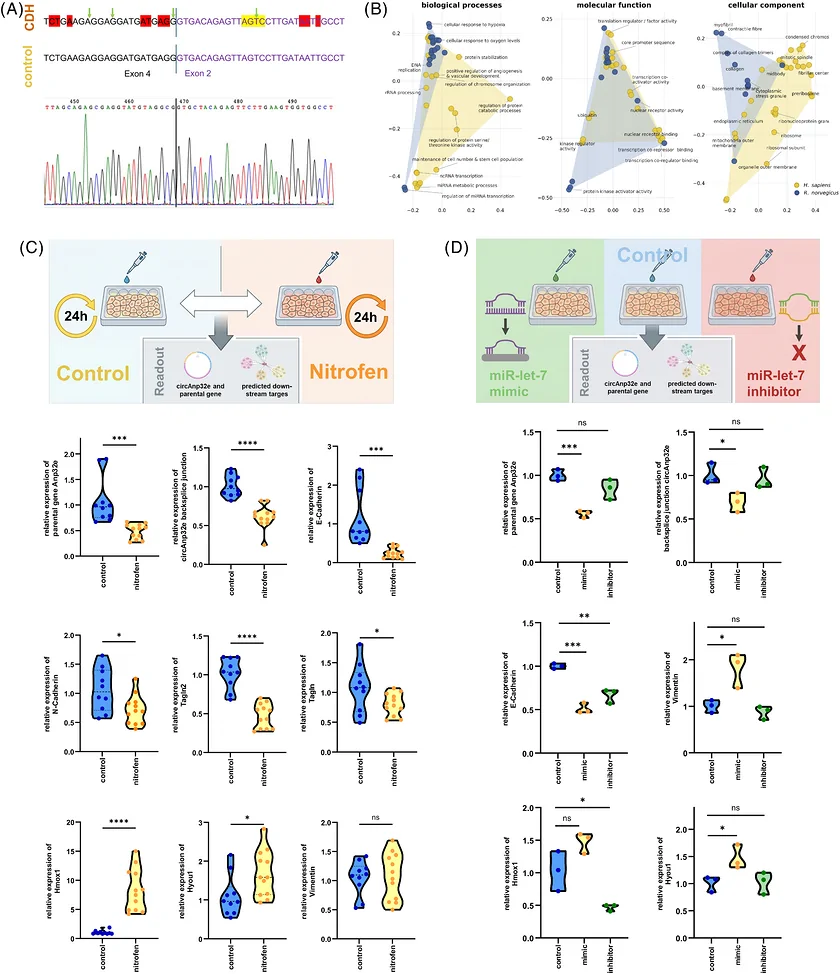
Conserved circular RNA (circRNA) sequence and strong overlap in biological function (miRNA sponging) in rat and human congenital diaphragmatic hernia (CDH). The circAnp32e mature sequence is highly conserved between the two species. Sanger sequencing of the circAnp32e back‒splice junction in formalin‐fixed, paraffin‐embedded human lung (*n* = 3 CDH patients and *n* = 3 controls). Base differences in three CDH and control sequences are annotated (substitutions = red; insertions = green; deletions = yellow) (A). We compared the circRNA profile in human CDH lungs at mid‐ and end‐gestation (*n* = 6; re‐analysed from Wagner et al.^5^) with the findings in our nitrofen rat model. There was a strong overlap for the Gene Ontology (GO) terms from predicted circRNA::miRNA::mRNA downstream targets between the two species; in particular for binding, RNA processes, response to hypoxia and vascular development (B). Next, BEAS‐2B cells were exposed to nitrofen or vehicle (DMSO) and analysed by RT‐qPCR. Relative expression of circANP32e, its parental gene *ANP32e* and CRAFT‐predicted downstream targets was determined (*n* = 10‒12; C). Cells were exposed to a let‐7b‐5p mimic or inhibitor (25 µM) or a non‐targeting control and RT‐qPCR of circANP32e, its parental gene *ANP32e* and CRAFT‐predicted downstream targets were performed (*n* = 3; D).

Lastly, we evaluated the human orthologue of circAnp32e for comparative analysis. Rat circAnp32e(2,3,4).1 (or rno‐Anp32e_0001) aligned 90% with human circANP32E(2,3,4).1 (hsa‐ANP32E_0007) (Figure S5D). PCR amplification and Sanger sequencing successfully confirmed its expression in human foetal lungs (Figure 5A). CDH samples carried more base changes than controls, but FFPE‐derived, low‐abundance templates are prone to sequence artefacts and these differences should be interpreted as hypothesis generating rather than disease specific. Furthermore, both orthologues are predicted to sponge similar miRNAs (Figure S6D).

### 3.6 Functional validation of the circANP32E::let‐7::mRNA target axis in a human bronchial epithelial nitrofen‐injury model

To provide functional support for the predicted circANP32E network in a human epithelial context, we exposed human bronchial epithelial cells (BEAS‐2B) to nitrofen and profiled circANP32E and its CRAFT‐predicted downstream targets. In line with the downregulation observed in late‐gestation (E21) rat CDH lungs, nitrofen exposure significantly reduced expression of circANP32E (*p* = .011) and its parental gene *ANP32E* (*p* = .018) at 24 h (Figure 5C). We next quantified CRAFT‐predicted targets of let‐7b‐5p and let‐7e‐5p (*HMOX1*, *HYOU1*, *TAGLN*, *TAGLN2*, *E‐CADHERIN* and *VIM*). *E‐CADHERIN* was downregulated (*p* = .007 at 24 h), as was *TAGLN and TAGLN2* (*p* = .048 and *p* = .007 at 24 h; Figure 5C). Conversely, Heme oxygenase‐1 (*HMOX1*), a canonical effector of the cellular response to hypoxia and oxidative stress, and hypoxia‐upregulated chaperone *HYOU1* (*p* = .02) were upregulated in nitrofen‐exposed BEAS‐2B (*p* = .002 at 24 h; Figure 5C). *VIM* (*p* = .88 and .71) was unchanged (Figure 5C). These changes tie the predicted circANP32E::let‐7 network to the hypoxia‒response pathways enriched in rat (Figure 4A) and human CDH.

To move from correlation toward function, we directly exposed BEAS‐2B cells to miR‐let‐7b‐5p (Figure 5D). The let‐7b mimic significantly and reproducibly reduced both *ANP32e* and circANP32E at 25 µM (*p* = .0006 and .03). The inhibitor had no effect, indicating that added let‐7b activity can repress ANP32E but endogenous let‐7b does not restrain it at baseline. We confirmed that miR‐let‐7b modulated the expression of the predicted downstream targets: *E‐CADHERIN* decreased (mimic *p* = .0002; inhibitor *p* = .003), *Vimentin* increased (mimic *p* = .02), *HYOU1* increased (mimic *p* = .04) and HMOX1 decreased upon let‐7b inhibition (*p* = .03). Notably, the let‐7b mimic reproduced the expression patterns of circANP32E, its parental gene, *E‐CADHERIN* and *HYOU1* seen in nitrofen‐injured epithelium. However, we did not observe a uniform, unidirectional target de‐repression predicted by a simple miRNA sponge function, suggesting that circANP32E acts within a broader let‐7‐responsive network.

## 4 DISCUSSION

The aetiology of CDH remains unknown but probably reflects genetic susceptibility and environmental insults that perturb epigenetic modifications affecting gene expression at critical developmental windows.^2^ Among these, DNA methylation,^17^ histone acetylation^18^ and non‐coding RNAs^5^, ^19^ have been implicated in CDH. Here, we determine the circRNA expression profile and its predicted miRNA‐sponging functions across early and late abnormal lung development and provide a first functional test in human epithelial cells. We identify a sex‐specific dysregulation of circAnp32e, circPpp3ca and circLarp4b, with spatiotemporal localisation of circAnp32e in the pulmonary epithelium. We demonstrate for the first time conserved circRNA forms and functions in the nitrofen rat model and human CDH. Our findings identify specific DE circRNAs, foremost circAnp32e, as candidate biomarkers and mechanistic contributors to abnormal lung development in CDH.

Although circRNAs have been characterised during normal rodent lung development,^20^, ^21^, ^22^ we describe a distinct circRNA biosignature in CDH‐associated abnormal lung development. Consistent with the foetal enrichment and spatiotemporal specificity of circRNAs,^23^, ^24^ our data reveal pronounced developmental dynamics. At E15, individual parental genes gave rise to a broader range of circRNA isoforms in keeping with circRNA regulation of cell proliferation, differentiation and lineage specification in early development. As development progresses, this per‐gene diversity narrowed even as the number of DE circRNAs strongly increased (119 at E15 vs. 681 at E21; Figure S1F), predicted targets shifted towards specialised functions related to tissue maturation and organogenesis, including environmentally responsive programs such as inflammation and hypoxia (Figure 4A,B). Nanopore sequencing revealed a 1.7‐fold increase in total transcripts from E15 to E21, and an additional 1.3‐fold increase after nitrofen exposure. A similar developmental accumulation of circRNAs alongside progressive refinement has been reported during porcine brain development.^25^ We interpret the loss of isoform diversity as RBP‐directed stabilisation that concentrates its regulatory role onto fewer, more critical isoforms as differentiation proceeds. *Ppp3ca*, for example, yielded 20 circRNAs at E15 but only four at E21, one of them dysregulated in CDH. Recurrent chromosomal circRNA ‘hotspots’ (Figure S5C) further suggest cis‐ and epigenetically regulated loci whose dysregulation may overlap disease‐associated regions.^26^

At both stages, predicted circRNA targets converged on inflammation, hypoxia and epithelial/mesenchymal/smooth muscle cell proliferation corroborated by Nanopore sequencing. Abnormal pulmonary remodelling in CDH is driven in part by EMT and endothelial‐to‐mesenchymal transition (EndoMT)^2^, the transition of epithelial and endothelial cells to mesenchymal cell‐like phenotypes (e.g., myofibroblasts and smooth muscle cells) under physiological and pathological conditions.^27^ Elevated inflammatory signalling in CDH may facilitate EMT and EndoMT, promoting pulmonary fibrosis and vascular remodelling.^2^, ^28^, ^29^, ^30^ Depending on their targets, circRNAs may modulate inflammation, apoptosis, fibroblast proliferation and extracellular matrix deposition.^31^ For example, during transforming growth factor‐beta (TGF‐β1)‐induced EMT, circRNAs are dynamically regulated by splicing factors like Quaking,^27^, ^32^ which is dysregulated in CDH lungs.^33^ Several dysregulated circRNAs at E21 derive from established drivers of EMT and EndoMT, including *EZH2*, *Akt3*, *Clasp2*, *EP300* and *PTK2*.^27^, ^34^, ^35^ TGF‐β1 itself is a principal driver of pulmonary vascular remodelling and a canonical inducer of EndoMT. Gong et al. recently showed that TGF‐β1 drives EndoMT and angiogenic behaviour in haemangioma‐derived endothelial cells in vitro and in vivo.^36^ A direct role for circRNAs in EndoMT has independent precedent. In brain endothelium after stroke or inflammatory stimulation^37^, ^38^ and in human umbilical vein endothelial cells subjected to sera from patients with Kawasaki syndrome^39^, ^40^ circRNAs drive transition through miRNA sponging, regulating tight junctions, interleukins and mesenchymal markers. Among our candidates, increased circAnp32e expression in the epithelium of nitrofen‐induced lungs led us to speculate on a regulatory role for this specific circRNA in CDH‐associated EMT.

Moreover, these relationships appear conserved across species: circAnp32e shared (1) its mature sequence, (2) its predicted miRNA interactions between rats and humans (Figure S6D), and (3) downstream targets enriched for hypoxia, transcription/translation regulation and inflammatory/infectious pathways (Figure 5B). Despite strong associations, this evidence remains correlative and the definitive functional role of specific circRNAs in EMT and EndoMT require direct testing. Our data suggest circAnp32e as a candidate for future mechanistic studies.

The recurrent hypoxia signature (Figure 4A) aligns with a growing literature placing circRNAs upstream of the hypoxia‐inducible factor (HIF) axis, for example, hsa_circ_0081065 sponges miR‐665 to raise HIF‐1α and aggravate hypoxia‐induced EndoMT.^41^ Within this framework, the predicted circANP32E::let‐7 interaction is coherent through two complementary routes. On the miRNA end, let‐7 regulates EMT‐ and fibrosis‐related factors in the alveolar epithelium: TGF‐β1 represses let‐7d to de‐repress *HMGA2* and mesenchymal markers in alveolar epithelium.^42^ Hence, circANP32E‐dependent changes in let‐7 availability could modulate this program, although the net direction may be context dependent (see functional data). On the chromatin level, ANP32E is the dedicated H2A.Z chaperone that controls the variant's deposition and removal from chromatin,^43^ with additional roles in regulating H2A.Z stability^44^ and H2A.Z removal is required for the TGF‐β‐induced EMT switch.^45^ Therefore, altered ANP32E dosage could reshape EMT‐associated chromatin, consistent with the pro‐fibrotic activity of ANP32E.^9^ Interestingly, these parts are linked: ANP32E is itself a validated let‐7c target in lung adenocarcinoma cells,^46^ indicating that circAnp32e, its parental transcript and let‐7 form an interlinked module rather than a linear sponge. We confirm this repression functionally: a let‐7b mimic reduced both *ANP32E* and circANP32E. That let‐7b‐5p and let‐7c‐5p rank among the circulating miRNAs associated with pulmonary hypertension and chronic lung disease in human CDH lends clinical weight to this axis.^16^

As a first functional test, we exposed human bronchial epithelial (BEAS‐2B) cells to nitrofen. This reproduced the late‐gestation downregulation of *ANP32E* and circANP32E seen in rat CDH lungs and produced a partial, stress‐driven loss of epithelial identity rather than a completed mesenchymal switch: *E‐CADHERIN* and both transgelins (*TAGLN* and *TAGLN2*) decreased, *VIM* was unchanged and the stress‐responsiveeffectors *HYOU1* and *HMOX1* were induced. To investigate the miRNA‐let‐7 mechanism directly, we stimulated the BEAS‐2B cells with a let‐7b‐5p mimic or inhibitor. The mimic decreased both *ANP32E* and circANP32E at 25 µM, whereas the inhibitor did not. This provides direct functional support for the reciprocal *ANP32E* –let‐7 circuit inferred above,^46^ in which let‐7 is both sponged by and represses the *ANP32E* locus. Several predicted downstream targets were let‐7b‐responsive (*E‐CADHERIN*, *VIM*, *HMOX1* and *HYOU1)*, but not as a linear sponge predicts: the mimic decreased *E‐CADHERIN* and raised *VIM* (opposite to the canonical epithelial‐protective role of let‐7), *HMOX1* was downregulated with let‐7 inhibition rather than tracking circANP32E, and *TAGLN* was unaffected by either. Together with the strong *HMOX1* induction under nitrofen, these patterns place circANP32E within a broader oxidative stress and EMT‐associated network in epithelial cells. Establishing causality and direction will require future direct circANP32E gain‐/loss‐of‐function together with luciferase reporter and miRNA pull‐down assays in epithelial and organoid models.

Our validated candidates diverge in expression from their linear counterparts, as reported in human foetal brain and rat lung development.^20^, ^47^ ANP32E, PPP3CA, TIAL1 and LARP4B are linked to transcriptional regulation, apoptosis and chromatin modification: ANP32E is a conserved histone chaperone enriched in cardiac endothelial cells and implicated in the life cycle of various viruses.^10^, ^44^, ^48^ PPP3CA is a calcineurin subunit involved in cardiac remodelling, T‐cell activation and cell cycle control/apoptosis.^12^, ^49^ Lastly, LARP4B and circLarp4b are involved in mRNA metabolism and regulators of proliferation and migration in cancer.^50^, ^51^, ^52^ LARP4B and TIAL1 are RBPs and act in a regulatory network to modulate mRNA turnover and translation.^6^ Furthermore, some RBPs auto‐regulate their own circRNA production^53^, ^54^ possibly explaining the opposite expression dynamics of the parental genes *Tial1* and *Larp4b* and their respective circRNAs.

Others have reported enrichment in clusters associated with biological functions of the parental genes for organ‐specific circRNAs.^23^ circRNAs are enriched in cardiac remodelling^55^, ^56^ during stress responses^35^ and in the fibrotic lung.^31^ We observed clustering of circRNA::miRNA::mRNA interactions and associated parental genes in several biological processes, including mRNA processing, development and enzyme/RNA binding at E15, and smooth muscle cell proliferation at E21. Additionally, circAnp32e shared common miRNAs with other circRNAs, including circPpp3ca, whose targets augmented apoptosis‐related pathways, smooth muscle cell proliferation regulation and miRNA transcription. Our results suggest coordinated regulatory networks formed by circRNAs, miRNAs and mRNAs, that involve parental gene expression.

CDH is more prevalent in males who also seem to be more susceptible to adverse pulmonary outcomes (including unpublished data from our group). Our sex‐disaggregated analysis revealed male‐specific upregulation of circAnp32e at E15, followed by general downregulation at E21. CircLarp4b was similarly elevated in male CDH lungs, but not being significantly dysregulated overall (Table 1). This earlier, male‐predominant dysregulation may contribute to a less constrained response to hypoxia and inflammation, consistent with the greater pulmonary morbidity of male CDH patients. Similar results have been reported in rat brain and lung development.^20^ No DE circRNAs mapped to the Y chromosome in rat or human CDH, but estradiol‐responsive pathways enriched downstream targets at E21, indicating a potential role for sex hormones. Accumulating evidence suggests sex‐specific differences in organ structure, development and function, which may be partially explained by variations in transcription factor binding, with 37% of all genes exhibiting sex differences in at least one tissue.^57^, ^58^ Further, sex‐specific regulation of RBP activity or upstream splicing mechanisms may influence circRNA biogenesis independently of parental gene expression. Supporting this, circRNAs have been shown to regulate miRNA biogenesis in a sex‐specific manner in neonatal lung conditions such as bronchopulmonary dysplasia.^53^

While circRNA‐based therapies have been explored in cancer,^59^ prenatal application studies remain limited due to potential off‐target effects and safety concerns. Their stability and stage‐ and tissue‐specific expression support their use as non‐invasive prenatal biomarkers.^5^, ^6^, ^60^ Recently, our team profiled circRNAs in amniotic fluid from CDH pregnancies undergoing fetoscopic endoluminal tracheal occlusion and identified 514 DE circRNAs distinguishing survivors from non‐survivors, of which 24 overlapped with our end‐gestation human CDH lung biosignature.^5^, ^60^ Two candidates (circFOXP1 and circSMARCC1) were confirmed by back‒splice junction sequencing directly in amniotic fluid.^60^ These findings indicate that tissue circRNA landscapes are recoverable from clinically accessible prenatal biofluids and justify the prospective evaluation of circRNAs as prenatal biomarkers.

### 4.1 Limitations

Microarrays enable high‐throughput detection of circRNAs through back‒splice junctions, including in low‐input FFPE tissue and biofluids, but depend on pre‐designed probes and thus, miss novel or low‐abundance circRNAs. Furthermore, our functional inferences relied on bioinformatics prediction, which is particularly challenging beyond human and mouse. Alternatively, Illumina RNA sequencing provides broader circRNA identification and parental gene expression data but is limited by low read coverage, short read lengths, accurate quantification due to low abundance, and intricacies of complex bioinformatics analyses.

circRNA detection and quantification remain technically demanding due to relatively low expression levels and potentially high false‐positive rates.^22^ Therefore, we performed targeted amplification using samples with and without RNase R treatment. RT‐qPCR and in situ hybridisation validation were limited for circPpp3ca and circLarp4b, whose high Cq values and weak signal indicate genuinely low copy numbers rather than probe failure. We do not claim that circPpp3ca or circLarp4b individually exert strong miRNA‐sequestering activity. Our network analysis predicts that circPpp3ca shares targets with the abundant circAnp32e (Figure 4C,D), raising the possibility that co‐expressed circRNAs act cooperatively on a shared miRNA pool.

Human circANP32e was sequenced from FFPE tissue, a recognised source of C > T/G > A deamination artefacts at low template input. Therefore, the base differences between CDH and control require confirmation in fresh‐frozen tissue before they can be considered disease specific. Finally, although the BEAS‐2B model provides functional, human‐cell support for dysregulation of circAnp32e and predicted downstream targets, this immortalised monoculture does not capture the cellular complexity of the foetal lung. Future studies using direct circRNA::miRNA binding assays and primary or organoid models are required to establish causality.

## 5 CONCLUSION

We show distinct circRNA biosignatures for abnormal lung development in our model of CDH with suggested conserved elements across rat and human CDH. circRNA‐mediated miRNA sponging appears related to inflammation/immunity and regulation of epithelial/mesenchymal/smooth muscle cell proliferation. Our data imply that circRNAs could constitute a mechanistic link between genetic predisposition and environmental insults, and may serve as prenatal biomarkers for timely detection of abnormal lung development.

## AUTHOR CONTRIBUTIONS

*C* *onception, data acquisition/analyses/interpretation and drafting the manuscript*: Marietta Jank and Arzu Öztürk Aptekmann. *Data acquisition/analyses/interpretation*: Rina Tanaka and Richard LeDuc. *Data analyses*: Muntahi Mourin. *Data acquisition*: Nolan De Leon, Daywin Patel, Claire McCallum, Richard Wagner, Shana Kahnamoui and Yuichiro Miyake. *Conception and data acquisition/analyses*: Matthew Kraljevic. *Conception and data acquisition*: Wai Hei Tse. *Data acquisition/analyses*: Athanasios Zovoilis. *Funding and revising the manuscript*: Takashi Doi and Michael Boettcher. *Conception, data acquisition/analyses/interpretation, funding and revising the manuscript*: Richard Keijzer.

## CONFLICT OF INTEREST STATEMENT

The authors declare they have no conflicts of interest.

## ETHICS STATEMENT

Procedures were approved by the Animal Research Review Committee (23‐026 [AC11831]) and Human Research Ethics Board (HS15293 [H2012:134]) at the University of Manitoba.

## Supporting information



Supporting Information

Supporting Information

Supporting Information

Supporting Information

## Data Availability

The datasets used in this study are deposited in public repositories. Rat circular RNA microarray data and Oxford Nanopore direct RNA sequencing data have been submitted to the Gene Expression Omnibus (accession numbers GSE346216, GSE346219 and GSE344409). Publicly available datasets used in this study include the human CDH circRNA microarray data (Wagner et al.^5^) and the circAtlas database entries for rno‐Anp32e_0001 and rno‐Ppp3ca (https://ngdc.cncb.ac.cn/circatlas/, accessed 28.7.2024). The modified CRAFT pipeline and the FindBacksplice tool used for circRNA prediction and back‒splice junction localisation are publicly available as described previously^8^ and at https://github.com/m‐kraljevic/modified‐CRAFT‐circRNA‐CDH‐pipeline.
