# Supplementary material for: Dysregulated circRNA expression profile and associated miRNA sponging in abnormal lung development in congenital diaphragmatic hernia
Source: Clin Transl Med. 2026 Sep 11;16(9):e70752. doi: 10.1002/ctm2.70752 (PMC13563360; doi:10.1002/ctm2.70752)
Supplement: Supplementary file 1 [file CTM2-16-e70752-s002.docx]

**Dysregulated circRNA expression profile and associated miRNA sponging in abnormal lung development in congenital diaphragmatic hernia**

Marietta Jank, Arzu Ozturk Aptekmann, Rina Tanaka, Muntahi Mourin, Nolan De Leon, Matthew Kraljevic, Daywin Patel, Wai Hei Tse, Claire McCallum, Richard Wagner, Shana Kahnamoui, Yuichiro Miyake, Athanasios Zovoilis, Takashi Doi, Michael Boettcher, Richard LeDuc, Richard Keijzer

**ONLINE DATA SUPPLEMENT**

**Supplementary Methods**

We investigated circular RNA (circRNA) dysregulation in the nitrofen rat model of congenital diaphragmatic hernia (CDH) at embryonic days (E)15 and E21 using an integrated experimental–computational workflow, followed by cross-species comparison with human CDH. For discovery, three nitrofen and three control foetal left lungs per time point were profiled on *Arraystar Rat Circular RNA Microarrays* after RNase R enrichment. Raw data were processed with Agilent Feature Extraction and R, and differential expression was assessed with a linear model and Benjamini–Hochberg correction (adjusted *p* < 0.05). Back-splice junction coordinates for differentially expressed (DE) probes were mapped to the rat genome using *FindBacksplice*.^1^ Using these genomic coordinates, we generated predicted mature circRNA sequences with a modified CRAFT pipeline, identified miRNA binding sites (miRanda; score > 140, energy < −20), and compiled candidate mRNA targets via multiMiR.^2^ Functional enrichment of DE parental genes and predicted circRNA::miRNA::mRNA targets employed GO and KEGG. We corroborated these results using Oxford Nanopore RNA sequencing. After preprocessing, we tested differential expression with two-sided t-tests and Benjamini–Hochberg correction, then compared observed DE genes to predicted circRNA::miRNA::mRNA targets and repeated GO/KEGG analyses to confirm pathway-level concordance.

For molecular validation and quantification, total RNA and genomic DNA were isolated with on-column DNase/RNase steps. We confirmed circRNA circularity by divergent primer PCR across BSJs on RNase R–treated cDNA, pJET subcloning, and Sanger sequencing (directional PCR to verify internal exon order where applicable). RT-qPCR quantified circRNAs and their parental mRNAs from the same cDNA pool. Spatial localization was assessed by BaseScope™ in situ hybridization using target-specific probes.

Finally, we performed a cross-species comparison by aligning rat circRNA findings (including the circAnp32e mature sequence) and enriched pathways with previously published human CDH circRNA microarray datasets at mid- and end-gestation^3^, highlighting conserved sequence features and biology. Experimental details and analysis code are provided in the following section, as well as in Tables S1–S5.

# *Circular RNA Microarray*

Arraystar Rat Circular RNA Microarrays were performed on n=3 control and n=3 nitrofen-induced biological replicates per time point (E15 and E21) and each replicate was a pool of 4-5 foetal lungs from a single dam. RNA was extracted (RNeasy Kit, Ref. 74104, Qiagen), quantified and quality assessed (NanoDrop ND-1000, Thermo Scientific), and checked for integrity by denaturing agarose gel electrophoresis. CircRNAs were enriched by RNase R digestion (Epicentre, Inc.), labeled using random primers (Arraystar Super RNA Labeling Protocol), hybridized to Arraystar Rat Circular RNA Microarrays (Ref. AS-S-CR-R-V2.0, Arraystar Inc.), incubated at 65°C for 17h, and scanned on an Agilent Scanner G2505C. Data were processed using Agilent Feature Extraction Software (v11.0.1.1) and R. Detection thresholds were determined via background signal slope analysis.

# *Bioinformatics analysis and modified CRAFT*

Differential expression analysis of the circRNA microarray expression data was performed using a linear model as implemented by the *lm()* function, and Benjamini-Hochberg multiple testing correction performed with *p.adjust()*, both in the R package, *stats*, version 4.4.1. circRNAs with a nominal p-value less than 0.05 were considered differentially expressed (DE) and circRNAs surviving Benjamini-Hochberg correction at a false discovery rate of less than 0.05 were designated as FDR-corrected DE circRNAs.. Observations with incomplete data were excluded from analysis.

Back-splice coordinates of DE circRNAs were generated using our published algorithm *FindBacksplice*.^1^ To provide downstream circRNA::miRNA::mRNA target prediction, the CRAFT pipeline was modified to fit system requirements and tool availability. Putative circRNA mature sequences were produced from the CRAFT script *script_sequence_extraction.sh*. MicroRNA binding sites were predicted with miRanda v3.3 (pairing score >140, energy <−20) as implemented in the CRAFT script *script_miRNA_detection.sh*. Downstream targets were identified with the R library multiMiR version 1.26.0.

Finally, we performed GO and KEGG pathway analyses of DE parental genes and circRNA::miRNA::mRNA targets as well as on the Oxford Nanopore RNA sequencing datasets. Pathway analysis was carried out in R v4.4.1 with the libraries KEGGREST v1.44.1 and GO.db v3.19.1, representing a snapshot of the Gene Ontology on January 17, 2024.

The corresponding code available on our public GitHub repository at <https://github.com/m-kraljevic/modified-CRAFT-circRNA-CDH-pipeline>

# *RNA isolation, RNase R treatment, and DNA isolation*

RNA was extracted from fetal lungs at E15 and E21 using PureLink™ RNA Mini Kit (Ref. 12183018A, Invitrogen) by on-column DNase I treatment (Ref. 12185010, Invitrogen). RNA was quantified (NanoDrop ND-1000) and visualized on a 2% bleach agarose gel. To enrich for circRNAs, 2–4 μg RNA was digested with RNase R (25U; Ref. RNR07250, Lucigen) by incubating at 65°C (5–10 min), 37°C (20 min), and 70°C (10 min). Genomic DNA was isolated using PureLink™ Genomic DNA Mini Kit (Ref. K1820-01, Invitrogen) with in-column RNase A digestion.

# *cDNA library generation, circRNA backsplice-junction detection, subcloning, and Sanger sequencing*

cDNA synthesis was performed with Maxima H Minus Reverse Transcriptase (Ref. EP0751, Thermo Scientific). Primers were designed via Primer3Web (version 4.1.0; Table E2). PCR was carried out with 2X DreamTaq PCR Master Mix (Ref. K1071, Thermo Scientific™) on a Veriti™ 96-Well Thermal Cycler (Applied Biosystems). Directional PCRs used Q5 High-Fidelity DNA Polymerase (Ref. M0491S, NEB) on 1:10 or 1:20 diluted RNase R-treated cDNA. PCR products were separated on 2.5% agarose gels, stained with SYBR™ Gold Nucleic Acid Gel Stain (Ref. E11494, Invitrogen), purified (PureLink™ Quick Gel Extraction Kit, Ref. K210012, Invitrogen), and subcloned with CloneJET PCR Cloning Kit (Ref. K1232, Thermo Scientific™). Ligation products were transformed into NEB 5-alpha Competent E. coli (Ref. C2988J, NEB) and selected on LB-ampicillin plates. Positive colonies were expanded, after which plasmids were extracted (GeneJET Plasmid Miniprep Kit, Ref. K0502, Thermo Scientific™) and sequenced (TCAG, SickKids, Toronto). Sequences were analyzed with ApE, plasmid editor ^8^ and 4Peaks software (Nucleobytes B.V., Aalsmeer, The Netherlands).

# *RT-qPCR*

Following primer optimization and sequence verification, RT-qPCR was performed with SsoAdvanced Universal SYBR Green Supermix (Ref. 1725271, BioRad). cDNA was diluted 1:100 for parental genes and 1:10–1:20 for circRNAs. Expression was normalized to GAPDH using the ΔΔCt method. Details are provided in Table E2.

# *Oxford Nanopore sequencing*

RNA libraries were prepared using the direct RNA sequencing kit (SQK-RNA004) (Oxford Nanopore Technologies) following the recommended protocol. A PromethION 24 device was used for sequencing with Flow Cells (FLO-PRO004RA). The flow cells were thawed at room temperature for 20 minutes, and available pore numbers were checked prior to loading the libraries. For detailed protocol of the Oxford Nanopore sequencing platform see Table S5.

A total of 107,058,591 long read RNA sequences were obtained, distributed as E15 Nitrofen 1 8856205, E15 Nitrofen 2 8855938, E15 Nitrofen 3 8871421, E15 Control 1 8853415, E15 Control 2 8849674, E15 Control 3 8853820, E21 Nitrofen 1 9071626, E21 Nitrofen 2 9163000, E21 Nitrofen 3 8863975, E21 Control 1 9077035, E21 Control 2 8881639, and E21 Control 3 8860843. The resulting sequences were assembled using StringTie. Long read sequencing negated the need to perform transcript-level inferences and RPKM and TPM values were calculated for each transcript. A two-sided t-test was performed in Python on the TPM values for each of the time points and Benjamini-Hochberg multiple testing correction was utilized. See Supplementary Table S9.

1.	Kraljevic M, Ozturk A, Jank M, LeDuc RD, Keijzer R. FindBacksplice: a Tool for Locating Circular RNA Backsplice Coordinates [Internet]. bioRxiv; 2025 [cited 2025 Sept 15]. p. 2025.09.08.674962. Available from: https://www.biorxiv.org/content/10.1101/2025.09.08.674962v1

2.	Dal Molin A, Gaffo E, Difilippo V, Buratin A, Tretti Parenzan C, Bresolin S, et al. CRAFT: a bioinformatics software for custom prediction of circular RNA functions. Brief Bioinform. 2022 Mar 10;23(2):bbab601.

3.	Wagner R, Jha A, Ayoub L, Kahnamoui S, Patel D, Mahood TH, et al. Can circular RNAs be used as prenatal biomarkers for congenital diaphragmatic hernia? European Respiratory Journal [Internet]. 2020 Feb 1 [cited 2022 July 21];55(2). Available from: https://erj.ersjournals.com/content/55/2/1900514

**Supplementary Figures**

**C**

**B**

**A**

**F**

**D**

**E**

**Supplementary Figure S1 – Unique biosignature for lung hypoplasia in CDH**

Control and nitrofen-induced lung samples at embryonic day (E)15 segregate based on their overall circRNA profile plotted on a principal component analysis (A). The heatmap reveals that CDH lung datasets cluster closely together indicating a similar circRNA profile. Among the controls, control lung 2 is more similar to the nitrofen group in its total and differentially expressed (DE) circRNA profile while controls 1 and 3 exhibit distinct clustering (B and C). Distribution of DE circRNAs at E15 resembles late gestation with the majority being exonic (77%) or sense overlapping (23%) (D). Numbers of circRNAs across all chromosomes and the ratio of circRNAs to the chromosome length reveal a similar pattern to E21 (see Figure 1G), with the highest circRNA enrichment on chromosomes 10, 8, and 12 (E). CircRNAs per parental gene at early and late gestation show a higher number of total circRNA isoforms per parental gene at E15 (F).

**A**

**B**

**Supplementary Figure S2 – Multiple circRNA isoforms derived from the parental genes *anp32e* and *ppp3ca* curated for the species rat by *circAtlas***

Investigation of multiple circRNA isoforms derived from the parental genes *anp32e* (A) and *ppp3ca* (B) curated for the rat species in the circAtlas database (accessed 11.11.2024). Only isoforms with back-splice junctions involving the same exons as our prime candidates/isoforms are shown.

**A**

down-regulated

up-regulated

differentially expressed

sufficient

reads

insufficient

reads

not

significant

**B**

**Supplementary Figure S3 – Detection of linear host genes *anp32e*, *ppp3ca* and *larp4b***

RNA sequencing was used to assess expression of parental genes of circRNAs: 57% of all parental genes at E21 were detected by Oxford Nanopore sequencing and 30% were DE (A), including *Tial1* confirming our previously published qPCR data. *Anp32e* appeared to be downregulated, but did not reach statistical significance likely due to small sample size (N=3 control and N=3 nitrofen-induced CDH lungs) (B).

**C**

**B**

**A**

**Supplementary Figure S4 – Enrichment for pathways related to hypoxia response, cell proliferation, vascular processes, and inflammation in circRNA::miRNA::mRNA downstream targets at E15**

The parental genes of DE circRNAs at E15 were enriched for pathways related to splicing/mRNA processing, and (mesenchymal, stem cell and neural crest) cell development as well as migration. Augmented molecular functions mainly involved binding activity, while impacted cellular components comprised membrane structures as well as complexes regulating gene expression and RNA processing (A). Next, we investigated potential biological function of circRNAs in early stages of abnormal lung development by circRNA::miRNA::mRNA interaction. The 730 target genes displayed increased enrichment for (smooth muscle, epithelial and endothelial) proliferation and migration, response to hypoxia, and vascular development (B). At E21, parental genes were associated with GO terms related to muscle hypertrophy and neural development/synapses (C).

**B**

**D**

**C**

**A**

**Supplementary Figure S5 – circRNAs in human CDH and highly conserved mature circAnp32e sequence across species**

The ratio of total circRNA number to the chromosome length at mid- (A) and end-gestation (B) showed the highest enrichment for chromosomes 17 and 19. Chromosomal locations of differentially expressed (DE) circRNA in rat and human CDH were plotted to detect potential “hot spots” of splice sites resulting in circRNA formation with red being mid-gestation/E15 and black end-gestation/E21 (C). Alignment of the circAnp32e mature sequence between the rat and human orthologues indicated a 90% sequence identity (D).

**A**

**cellular component**

**biological processes**

**molecular function**

**circRNA::miRNA::mRNA at mid-gestation**

**B**

**circRNA::miRNA::mRNA at end-gestation**

**C**

 **Rat circAnp32e	Human circAnp32e**

**D**

**Supplementary Figure S6 – Strong overlap in predicted biological function (miRNA sponging) of circRNAs between rat E21 and human CDH at mid- and end-gestation.**

Predicted circRNA::miRNA::mRNA at both mid- (A) and end-gestation (B) showed enrichment for biological processes such as RNA regulation, epigenetic pathways, and protein binding/modification in CDH lungs. In addition, KEGG pathway analyses revealed that downstream targets were associated with inflammation and infection (*e.g*., NF-κB and TNF signaling as well as viral infections), regulation of cell cycle and proliferation, and cardiovascular pathways. We observed a strong overlap in the top 15 enriched KEGG terms between rat E21 and human mid- and end-gestation CDH lungs (C). Further, human circAnp32e is predicted to sponge similar miRNAs to the rat circRNA equivalent shown in Figure 4C (D).

**D**

**C**

**B**

**A**

**cellular component**

**molecular function**

**biological processes**

**Supplementary Figure S7 – Confirmation of predicted KEGG pathways using the modified CRAFT pipeline and Oxford Nanopore Sequencing**

Gene Ontology (GO) analyses of Oxford Nanopore Sequencing revealed networks related to post-transcriptional regulation including mRNA splicing, mRNA processing, and histone modification (A). p-values of all KEGG terms derived from the modified CRAFT pipeline were plotted against the pathways enriched by RNA sequencing, resulting in a moderate positive correlation (r=0.38; B). DE genes between nitrofen-induced and control lungs detected by Oxford Nanopore Sequencing showed enrichment for KEGG terms related to infection/immunity, cell adhesion (tight junction and adherence junction), and cancer (C). The proportion of predicted genes, confirmed to be DE based on RNA sequencing, was visualized for each pathway (D).

**Supplementary Tables**

**Supplementary Table S1 – Sex determination: primers and protocol**

| **Primer/Protocol for sex determination** | |
| --- | --- |
| *Sry* forward | CCAGCATGCAGAATTCAGAGA |
| *Sry* reverse | CCCAGTCCTGTCCGTATATGAT |
| *Kdm5c* forward | AGACACCAGAGGCAGATAGA |
| *Kdm5c* reverse | CTCCATTAACAGGCGAGGAA |
| Protocol | Initial denaturation at 95°C for 2min;  Denaturation at 95°C for 30sec (35 cycles);  Annealing at 60°C for 30sec;  Extension at 72°C for 38sec;  Final extension at 72°C for 5min  The PCR products were analyzed by 1% agarose gel electrophoresis; the presence or absence of the *Sry* gene allowed for sex determination. |

**Supplementary Table S2 – Sequence validation: primers and protocols**

| **Primer** | **Sequence** | **Tm (°C)** | **product length** |
| --- | --- | --- | --- |
| *anp32e* forward | CAGGAGGACAATGAAGCGC | 58.89 | 158 bp |
| *anp32e* reverse | CCCTCTCCTACTTCTGAGCC | 58.59 |  |
| *circAnp32e* forward | ACGGATTTGATCAGGAGGACA | 58.46 | 156 bp |
| *circAnp32e* reverse | GGCCATACTGAGAAACTCCAG | 59.12 |  |
| *ppp3ca* forward | ACGTCCTGAACATCTGCTCA | 59.03 | 112 bp |
| *ppp3ca* reverse | CCTATTGCTCGGATCTTGTTCC | 58.87 |  |
| *circPpp3ca* forward | TGAGATGCTGGTAAACGTCCT | 58.73 | 131 bp |
| *circPpp3ca* reverse | GCTGCAGGAAGTCACATACAG | 59.65 |  |
| *Larp4b* forward | CAGAGCAGACAGACATGAGC | 59.08 | 104 bp |
| *Larp4b* reverse | CTGGCTTTCTGGTTGAGACTC | 59.25 |  |
| *circLarp4b* forward | AGAATACGAACCTCTGCCTGA | 59.11 | 154 bp |
| *circLarp4b* reverse | CAGATGAGAGCTGTCCTTGC | 59.1 |  |
| *IntAnp32e Ex 4* forward | TTGATCAGGAGGACAATGAAGC | 58.39 | 246 bp |
| *IntAnp32e Ex 3* reverse | GACTTCCAAGCCTCCAGAAATT | 58.57 |  |
| *IntLarp4b Ex-5* forward | AGCAGACAGACATGAGCACT | 59.02 | 249 bp |
| *IntLarp4b Ex-3* reverse | GAGTCAATGCTGGGAGGGAA | 59.38 |  |
| *GAPDH* forward | AAGAACCCTTCCGTAGCGA | 59.3 | 124 bp |
| *GAPDH* reverse | GCTCTCTGCTCCTCCCTGTTCTA | 58.9 |  |
| *h_Anp32e* forward | AGGAGGATGATGAGGATGGC | 58.64 | 166 bp |
| *h_Anp32e* reverse | TCCTCTTCTCCCTCTCCCAA | 58.91 |  |
| *h_Anp32e* forward | GAGGAAGAGGAGGATGAGGA | 57.23 | 151 bp |
| *h_Anp32e* reverse | CTTCCCCTTCTTCAACATAGTCA | 57.83 |  |
| *h_circAnp32e* forward | CAGGAGGATAATGAAGCGCC | 58.48 | 167 bp |
| *h_circAnp32e* reverse | CGGGCCAGCGAACTTAGTT | 60.38 |  |
| *h_E-cadherin* forward | CCACCAAAGTCACGCTGAATAC | 58.69 | 104 bp |
| *h_E-cadherin* reverse | GGAGTTGGGAAATGTGAGCAA | 59.31 |  |
| *h_Vimentin* forward | GAGAACTTTGCCGTTGAAGC | 58.04 | 120 bp |
| *h_Vimentin* reverse | GCTTCCTGTAGGTGGCAATC | 58.59 |  |
| *h_N-cadherin* forward | CCTCCAGAGTTTACTGCCATGAC | 58.31 | 149 bp |
| *h_N-cadherin* reverse | GTAGGATCTCCGCCACTGATTC | 58.99 |  |
| *h_Hmox1* forward | CCAGGCAGAGAATGCTGAGTTC | 59.23 | 144 bp |
| *h_Hmox1* reverse | AAGACTGGGCTCTCCTTGTTGC | 59.54 |  |
| *h_Hyou1* forward | CTTCAACCTGGATGAGAGTGGC | 58.83 | 128 bp |
| *h_Hyou1* reverse | ACAGGCTGGAAATGGTGTTGCC | 59.22 |  |
| *h_Tagln* forward | GTTCCAGACTGTTGACCTCTTT | 58.99 | 139 bp |
| *h_Tagln* reverse | CTGCGCTTTCTTCATAAACC | 59.23 |  |
| *h_Tagln2* forward | ATCCCAACTGGTTCCCTAAGAA | 58.99 | 92 bp |
| *h_Tagln2* reverse | CCCATCTGTAACCCGATCACG | 58.17 |  |
| *h_Gapdh* forward | GTCTCCTCTGACTTCAACAGCG | 59.94 | 131 bp |
| *h_Gapdh* reverse | ACCACCCTGTTGCTGTAGCCAA | 59.67 |  |

**Supplementary Table S3 – Probes for BaseScope^TM^ *in situ* hybridization**

| **Probe ID for circRNA detection and quantification** | |
| --- | --- |
| circAnp32e | NPR-0043351 |
| circPpp3ca | NPR-0043352 |
| circLarp4b | NPR-0042589 |

**Supplementary Table S4 – BaseScope protocol**

| **Step** | **Procedure** |
| --- | --- |
| **Sectioning** | 5 μm tissue sections mounted on Superfrost™ Plus slides (Fisher Scientific) |
| **Baking (1)** | Slides baked at 60°C for 1 h |
| **Deparaffinization** | Xylene (2×5 min), 100% ethanol (2×2 min) |
| **Baking (2)** | Slides dried at 60°C for 5 min |
| **Peroxide Treatment** | Hydrogen peroxide (ACD, Ref. 322335), 10 min at room temperature |
| **Water Wash & Acclimation** | Washed twice in distilled water, then 10 sec in distilled water at 100°C |
| **Target Retrieval** | 15 min at 100°C in RNAscope™ Target Retrieval Solution (ACD, Ref. 322000) |
| **Rehydration** | Slides in distilled water (10 sec), 100% ethanol (3 min) |
| **Baking (3)** | Slides baked at 60°C for 5 min |
| **Barrier Application** | Hydrophobic barrier drawn around each section |
| **Protease Treatment** | Protease III (ACD, Ref. 321710), 15 min at 40°C in HybEZ™ II oven |
| **Wash (1)** | RNAscope™ Wash Buffer (ACD, Ref. 310091), 2×2 min |
| **Probe Hybridization** | BaseScope™ probes applied for 2 h at 40°C in HybEZ™ II oven |
| **Amplification Steps** | Amplifier 1–6: 15–30 min at 40°C; Amplifier 7: 60 min room temperature; Amplifier 8: 30 min room temperature; Wash buffer 2×2 min between each step |
| **Detection** | Fast RED for 20 min at room temperature in the dark |
| **Counterstaining** | Gill’s hematoxylin (2 min), 0.02% ammonia water (2–3 dips) |
| **Baking (4)** | Slides dried at 60°C for 15 min |
| **Mounting** | VectaMount mounting medium (Vector Labs) |

**Supplementary Table S5 – Oxford Nanopore direct RNA library preparation and sequencing (SQK-RNA004)**

| **Step** | **Details** |
| --- | --- |
| **RTA ligation** | NEBNext® Quick Ligation Reaction Buffer (NEB B6058, 3 µL); RNaseOUT™ (Invitrogen 10777019, 1 µL); RTA (1 µL); T4 DNA Ligase (NEB M0202M, 1.5 µL); total RNA (1 µg). Incubate 10 min at room temperature. |
| **Add RT master mix** | To adaptor-ligated RNA add 23 µL master mix: nuclease-free water 9 µL; 10 mM dNTPs (NEB N0447) 2 µL; 5× First-Strand Buffer 8 µL; 0.1 M DTT 4 µL. Mix well. |
| **Add RT enzyme** | SuperScript™ III Reverse Transcriptase (Thermo Fisher 18080044), +2 µL per reaction; brief spin and mix. |
| **First-strand synthesis** | Thermocycler: 50 °C 50 min; 70 °C 10 min; hold 4 °C. |
| **Bead cleanup (post-RT)** | Mag-Bind® TotalPure NGS beads (Omega Bio-tek), 1.8× bead:sample; add 72 µL beads per RT mix; rotate 5 min; magnet 5 min (or until clear); wash 2× with 70% EtOH. |
| **Elute RNA–cDNA hybrids** | Elute in 23 µL nuclease-free water; resuspend pellet, rotate 5 min; elute on magnet. |
| **Sequencing adaptor ligation** | To 23 µL eluate add: NEBNext® Quick Ligation Reaction Buffer 8 µL; RNA Ligation Adapter (RLA) 6 µL; T4 DNA Ligase 3 µL. Incubate 10 min at RT. |
| **Final library cleanup & elution** | NGS beads 1× (40 µL); rotate 5 min; magnet 5 min; wash 2× with washing buffer; elute in 33 µL elution buffer. Use 1 µL for quant; 32 µL for sequencing (≤ 113 ng loaded). |
| **Device & flow cells** | PromethION 24; Flow Cells FLO-PRO004RA. Thaw at RT 20 min; check pore count; remove headspace air. |
| **Prime flow cells** | Load 500 µL priming mix (30 µL RNA flush tether + 1170 µL flow cell flush); after 5 min add another 500 µL. |
| **Prepare library mix & load** | Library mix 200 µL total: 32 µL final library + 100 µL Sequencing Buffer (SB) + 68 µL Library Solution (LIS). Load per ONT PromethION protocol. |
| **Sequencing** | Run up to 72 h; data collected as pod5 for downstream preprocessing. |

**Oxford Nanopore direct RNA sequencing quality control**

MultiQC report for E21

MultiQC report for E15

**Supplementary Table S6 – Excel tables of microarray results at E15** Lists only circRNAs that could be assigned to a parental gene. Intergenic circRNAs are not included. None were differentially expressed at E15.

**Supplementary Table S7 – Excel tables of microarray results at E21** Lists only circRNAs that could be assigned to a parental gene. Intergenic circRNAs are not included, which accounts for the difference from the DE counts in the main text. Fold changes in Table 1 of the manuscript are from the log_FC_raw column.

**Supplementary Table S8 – Strong overlap in the parental genes of circRNAs between rat and human CDH**

|  | **Overlap between mid- and end- gestation** | **Overlap between mid-gestation and E21** | **Overlap between end-gestation and E21** | **Overlap between mid-, end-gestation and E21** |
| --- | --- | --- | --- | --- |
| parental genes of all detected circRNAs | 4399 | 1055 | 883 | 882 |
| parental genes of DE circRNAs | 20  (*aff3, ambra1, anapc7, asap2, cntrob, col24a1, ddx17, dennd3, kcnh1, lmf1, lphn1, mkl1, pan3, phc3, pkd1l2, smyd3, tgfb1, trio, wdr37* and *zbtb40*) | 14  (*ambra1, bnip3l, dennd1a, erc1, eya3, nckap1, pan3, phc3, rapgef6, reep2, smyd3, spg7, susd1* and *zmym4*) | 31  (*abhd12, ambra1, arhgef12, armc8, cdc27, cdk14, clip2, cnot1, crim1, dtnb, eif4e3, ephb2, exoc4, hecw1, mapk1, pan3, pde4d, phc3, pkdcc, ppp3cc, ptpra, rtel1, scaf8, sec24a, slc4a7, smek2, smyd3, snx14, stk39, uso1* and *vps53)* | 4  (*ambra1, pan3, phc3* and *smyd3)* |
| parental gene function:   - Lung disease/ fibrosis/ SMCs | *mkl1, smyd3, tgfb1, ambra1* and *col24a1* | *bnip3l, smyd3,* and potentially *nckap1* | *ambra1, mapk1, pde4d, smyd3* and *ptpra* | *ambra1* and *smyd3* |
| - Embryonic development | *phc3* and *trio* | *eya3* and *phc3* | *ephb2* and *phc3* | *phc3* |
| - Cell proliferation: | *anapc7, cntrob, kcnh1, tgfb1* and *smyd3* | *ambra1, bnip3l, smyd3* and potentially *reep2* | *cdc27, cdk14, eif4e3, mapk1, pan3, ptpra* and *smyd3* | *ambra1, smyd3* and *pan3* |
| - RNA/DNA regulation: | *aff3, ddx17, pan3, phc3, smyd3* and *zbtb40* | *pan3, phc3, smyd3* and *zmym4* | *cnot1, eif4e3, pan3, phc3, smyd3, rtel1* and *scaf8* | *pan3, phc3* and *smyd3* |
| - Others: | *lphn1* (cellular adhesion) |  |  |  |

**Supplementary Table S9 – Excel tables of Sequencing results at E21**
